# Physical Activity Participation of Disabled Children: A Systematic Review of Conceptual and Methodological Approaches in Health Research

**DOI:** 10.3389/fpubh.2016.00187

**Published:** 2016-09-05

**Authors:** Samantha Mae Ross, Kathleen R. Bogart, Samuel W. Logan, Layne Case, Jeremiah Fine, Hanna Thompson

**Affiliations:** ^1^Social Mobility Lab, College of Public Health and Human Sciences, Oregon State University, Corvallis, OR, USA; ^2^Disability and Social Interaction Lab, School of Psychological Science, Oregon State University, Corvallis, OR, USA; ^3^College of Communication and Education, Chico State Autism Clinic, California State University, Chico, CA, USA

**Keywords:** assessment, disability, international classification of functioning disability and health, participation, physical activity, recreation and sport, systematic review

## Abstract

Physical activity (PA) participation is widely recognized as a critical component of health and development for disabled and non-disabled children. Emergent literature reflects a paradigm shift in the conceptualization of childhood PA as a multi-dimensional construct, encompassing aspects of physical performance, and self-perceived engagement. However, ambiguity remains around how participation as a health construct is integrated into PA research. The primary objective of the present mini-review is to critically examine current conceptual and methodological approaches to evaluating PA participation among disabled children. We conducted a systematic review of contemporary literature (published between 2000 and 2016). Seventeen articles met inclusion criteria, and their research approach was classified into guiding framework, definition of the key construct, and measurement used. The primary guiding framework was the international classification of functioning, disability and health. An explicit definition of PA participation was absent from all studies. Eight studies (47%) operationalized PA and participation as independent constructs. Measurements included traditional performance-based aspects of PA (frequency, duration, and intensity), and alternative participation measures (subjective perception of involvement, inclusion, or enjoyment). Approximately 64% of included articles were published in the past 2 years (2014–2016) indicating a rising interest in the topic of PA participation. Drawing from the broader discussion of participation in the literature, we offer a working definition of PA participation as it pertains to active, health-associated behaviors. Further description of alternative approaches to framing and measuring PA participation are offered to support effective assessment of health status among disabled children.

## Introduction

Engagement in moderate to high intensity physical activity (PA) during childhood is advocated for in the promotion of optimal health outcomes and may offset predisposed risk for the development of secondary health conditions experienced by disabled children (1–3). Participation in PA opportunities is a fundamental childhood experience that fosters the psychosocial development of interpersonal skills, self-confidence, and self-efficacy (4). Increased PA participation is a primary goal expressed by parents and professionals for disabled children (5–7). Given our focus on physiological and psychosocial health outcomes, we use the term *PA participation* in reference to “engagement in a physically demanding movement, sport, game, or recreational play that results in energy expenditure and perceptions of communal involvement” (8, 9).

A consistent understanding of the PA participation construct is necessary for key stakeholders to successfully describe the health status of disabled children. *Participation* is broadly conceptualized as “involvement in life situations” (10) within psychology and disability related literature, but ambiguity surrounds the intended meaning of the term (8, 9) as a measurable index of health relative to being physically active. Recent efforts to integrate this construct in health literature are exemplified by Kang and colleagues (11). For children who experience physical disabilities, they define optimal recreation and leisure participation as the quality of child–environment interactions reflected in individualized (objective and subjective) physical, social, and self-engagement outcome measures (p. 1735). Kang et al. (11) cautions against inferring *poor* health from observed *differences* in frequency and intensity of PA participation between disabled and non-disabled children, without consideration for quality of children’s experiences. Misperceptions about the extent to which a child can participate may result in fewer opportunities or expectations for disabled children and reduce engagement in this health-promoting behavior. Therefore, there is a critical need to further examine health indicators in an inclusive manner. The first step is to reach consensus on how PA participation is effectively discussed and measured as a health indicator for disabled children.

## Framing Physical Activity Participation

Physical activity has been traditionally discussed from a medical model framework in which health resides in the individual, represented by the absence of illness and body impairments. In response, PA has routinely been defined as “bodily movements that result in energy expenditure” (12). It is commonly operationalized as the frequency of activity attendance (13–16) or average daily time spent engaged at given intensity levels (e.g., light, moderate-to-vigorous) (17, 18). Subsequent attention has been given to identifying key activity restrictions or anatomical impairments, such as muscle weakness or low motor skill proficiency, to explain the limited PA engagement of disabled children (17, 19).

When using physiological performance outcomes as the single indicator of PA, there is an inherent assumption that functional deficits will inhibit disabled children from becoming “full” participants in community activities or sports teams. Reduced opportunities may limit a child’s exposure to fundamentally important physical, social, and personal experiences for health development. From an equity standpoint, additional qualifiers are needed to describe and appropriately measure PA patterns as a health index across disabled and non-disabled children. This requires a comprehensive discussion of both physical performance and psychosocial aspects of inclusion. For example, measures of self-concept (20), identity (4), and enjoyment (21) need to be considered alongside fitness and motor skill proficiency, to allow for more accurate and sensitive measurement of PA participation improvement among disabled children. Efforts to capture this multi-dimensional health aspect of PA for disabled children use the term “PA participation” [e.g., Ref. (11, 13)].

The term participation gained hold as a health indictor following the introduction of the International Classification of Functioning, Disability and Health (ICF) framework from the World Health Organization (WHO) in 2001 (10). The ICF reflects a shift away from the impairment-based disablement framework toward emphasis on the personal, social, and environmental impacts of disability on health (22). In contrast to the traditional medical model’s focus on individual attributes of health, this contemporary biopsychosocial model takes the perspective that disability occurs at a person-in-environment level. Within this framework, participation describes the extent to which a child is socially engaged in child-relevant life situations, such as organized and school-related sports, games, and recreational play with peers in the community (11, 23).

Ross et al. (23) published a guide for researchers to advance the study of childhood PA participation. Their guide includes three steps: (1) identify a health framework, (2) clearly define outcomes that operationalize PA within the context of a given research project, and (3) select appropriate PA measurements that map back onto the targeted conceptual dimension of health. As an extension of Ross et al. (23), the primary objective of the present mini-review is to critically examine current conceptual and methodological approaches to examining PA participation among disabled children. A systematic review of contemporary literature (published between 2000 and 2016), explicitly investigating PA participation as a health construct for disabled children, was conducted. The operationalized definition of this key construct and implemented measurement practices were evaluated to support our understanding of this phenomenon and inform future research efforts.

## Methods

A systematic review of contemporary literature (January 2000–January 2016) was conducted to examine the conceptualization and measurement practices for PA participation among disabled children. *Preferred Reporting Items for Systematic Reviews and Meta-Analysis* (PRISMA) standard guidelines were followed, as per recommended practice (24). An electronic database search was conducted in February 2016 and detailed in Appendix A in Supplementary Material.

### Initial Screening and Inclusion

Given strong links between PA participation and health among disabled children (2, 25), the primary objective of this study was to examine the use of this term in reference to active, health-associated levels of PA. The primary inclusion criterion was the use of the key terms “physical activity, sport, active, or recreation” in combination with “participation” as a measurable construct. Articles were excluded if the term PA referred to children’s leisure or more broadly defined participation outside-of-school or in daily life activities. Three trained research assistants independently screened titles and abstracts using this primary inclusion criteria, in addition to the following: (a) target population included children or youth, mean age ≤18 years, (b) must have included primary data other than case reports, (c) available in English, and (d) published in a peer-review journal. Exclusion criterion included (a) absence of the key words from the title, abstract, or the text body, (b) participants’ mean age was outside the target age range, (c) disabled children were not included as participants, or (d) the term “PA participation” was not used as a measurable outcome.

### Data Extraction and Synthesis

Articles retained after the initial screening underwent full review by three independent researchers. Data on study characteristics, key term definitions, and related measurement and methodology characteristics were extracted and synthesized. Any ambiguity around how the key term was used in an article was discussed among primary authors. The final data set was reviewed for emergent themes in the guiding framework, definition of key terms and assessment measures. A summary of the search and screening process can be found in Appendix A in Supplementary Material.

## Results

A total of 17 articles were included in this review (13, 26–41). Key study characteristics of the included articles are presented in Table 1. The majority of studies were published within the last 2 years (*n* = 11, 64% published 2014–2016), and most frequently published in *Research in Developmental Disability* (27, 33, 35, 36) or *Disability and Rehabilitation* (32, 34). Articles were primarily published in journals within psychology or medical related fields (e.g., *BMC pediatrics, Developmental Medicine and Child Neurology, Disability and Rehabilitation, Child: Care, Health and Development*). Public health and kinesiology journals, although the minority, were also represented in our sample (e.g., *Adapted Physical Activity Quarterly, Journal of Physical Activity and Health, and Journal of Sport and Health Sciences*). There were only one or two articles per year published between 2007 and 2013. Prior to 2007, only one article published in 2002 met inclusion criteria (41). The majority of the research was conducted in Canada (*n* = 5, 29%) and included participants aged 6–12 years (middle childhood, *n* = 11, 65%) who were representative of a broadly defined disability population (*n* = 5, 29%).

**Table 1:** **Key study characteristics of included articles. Includes (1) journal and country of publication; (2) Description of participant population (*n* = sample size; M = mean age in years of sample); (3) Key word pertaining to physical activity participation used and definition when provided; (4) Assessment and measure(s) used to capture the key construct; and (5) brief summary of associated results**.

Reference	Journal/country	Population (*n*, M age in years)	Key word and definition	Key word: assessment/measure(s)	Results
Arim et al. (26)	*International Journal of Pediatrics/*Canada	NDD/D and TD (*n* = 1,805/7,314, school aged)	*Participation in PA*: informal (unorganized) and formal (organized) PA were considered a separate types of PA	*Survey*: frequency of attendance in (a) organized sport or PA and (b) unorganized sport or PA in the last year; Dichotomized indicator of participation (0 = about once a month or never; 1 = about once a week or more)	NDD/D less likely to participation in organized sport or PA (~50% participated) compared TD, with 70% participation, after controlling for child and family factors. No significantly different participation rates between groups in unorganized sport or PA
					Age, but not sex, was associated with participation in PA

Ayvazoglu et al. (27)	*Research in Developmental Disabilities/*United States	ASD (*n* = 6, M = 7.5)	*Participation in PA; PA participation*: operational definition *not* provided	*RT3 Accelerometers*: number of minutes of MVPA per day	Low levels of PA as indicated by accelerometer data (M = 34.33 min/day MVPA)
				*Q-Sort of PA cue cards + Follow Interview*: rank Order and content analysis for emergent themes	Categories/*Themes*: (1) understanding PA in ASD: child (a) lacks social skills (b) is bullied or mocked (c) parental fear of being hurt (d) trouble transitioning; (2) living with a child with ASD: (a) lack of time, (b) too fatigued for PA, (c) external support (d) lack of money; (3) Awareness of ASD at school and community: (a) Why is child behaving that way? (b) Limited PA opportunity, (c) lack of disability knowledge, (d) teach PA skills (d) academics more important than PA

Bantjes et al. (28)	*International Journal of Disability, Development, and Education/*South Africa	CP (*n* = 15, M = 14.0)	*Participation in PA; participation in sport and exercise*: operational definition not provided	*Semi-structured, in-depth interview*: thematic analysis of the lived experiences of (1) range of involvement in PA and context of participation, (2) experience of participation in PA, (3) perception of factors that promote and hinder participation; and (4) ideas about factors that should be taken into account when developing programs	General consensus that there are limited number of sports and PA opportunities
					Themes about factors important for designing programs to promote [CP] participation in sport: (1) opportunities, variety, and choice; (2) adapted PA that take account of abilities; (3) autonomy and consultation; (4) friendship, social interaction, and belonging; (5) physical challenge and excitement; (6) coaching, progress, and mastery; (7) competition and opportunity to perform; (8) fairness and inclusion

Bloemen et al. (29)	*BMC Neurology/*Netherlands	SB (*n* = 33, M = 13.0)	*PA participation; Participation in PA*: “For this study PA consists of both PA in activities of daily life and participation in (un)organized sports. It is defined as ‘any bodily movement, produced by skeletal muscles, that results in energy expenditure’”	*Focus groups and interviews*: thematic analysis with inductive strategy to identify positive and negative PA determinants, classify determinants as personal or environment based on ICF, and specify a detailed description of the PA, positive, negative determinant, or solution	Personal factors: (1) intention, (2) attitude, (3) self-efficacy, and (4) health condition
					Environmental factors: (1) social/family influence and (2) facilitators and Barriers
					*See original manuscript for detailed list of sub-themes*

Capio et al. (30)	*Journal of Sport and Health Science/*Australia	CP and TD (*n* =24/26, M = 7.2)	*PA participation; PA level; PA engagement*: “…PA level represents a participation component [of the ICF]”	*Uni-axial Accelerometer*: % time LPA and MVPA	Weekday ≠ Weekend PA levels for CP and TD
					FMS training associated with significant increase in LPA and MVPA for children with CP, but not for typically developing (training × group effect)
					Increase in PA level associated with positive gains in FMS movement and skill patterns for locomotion

Harvey et al. (31)	*Physical Education and Sport Pedagogy/*Canada	ADHD (*n* = 10, M = 10.3)	*PA behaviors; PA participation; PA experiences*: operational definition *not* provided	*TGMD-2, MABC-2*: movement skill level	Children scored as very-poor to average in movement skills
				*Scrapbook-semi-structure interviews about PA experiences*: thematic analysis	Themes: (1) context (a) time and (b) environment, (2) play and types of PA engagement (a) organized activities, (b) leisure activities, (c) movement (i.e., function), and (d) positive outcome (e.g., social and enjoyment), (3) organization (a) constraints, (b) feelings, (c) how I learn, and (d) planning

Jaarsma et al. (32)	*Disability and Rehabilitation/*Netherlands	CWD, parents and health professionals *(n* = 30/36/17, M = 14.1)	*Sports participation*: “Sports participation falls under broader ICF term participation. Items of sports participation and disabilities were grouped according to components of TPB (attitude, subjective norm, perceived behavioral control)”	*Mail-Survey questionnaire*: type of sports participation	Almost all children participated in sports at school (96%) and after school (77%)
			[see original manuscript for definitions of each component]	*Semi-structured interviews*: thematic analysis of facilitator and barriers using ICF framework	Personal factors
					Barriers: (1) disability and (2) fatigue
					Facilitators: (1) health, (2) fun, (3) internal motivation, and (4) physical strength
					*Environmental*
					Barriers: (1) lack of facilities, (2) transportation, (3) dependency, (4) lack of acceptance, and (5) lack of information
					Facilitators: (1) social contacts, (2) support from family, (3) information, and (4) sports activities during school hours

King et al. (13)	*Child: Care, Health and Development/*Canada	CWD and TD (*n* = 781, school age)	*Participation in active PA; participation profile*: “Intensity of participation in active PA (e.g., doing team sports, racing or track and field)”	*Children’s Assessment of Participation and Enjoyment (CAPE)*^a^ *and Preferences for Activities of Children (PAC)*: dimensions of participation: diversity, intensity, with whom, where, and enjoyment of children’s participation in specific activity types (e.g., active physical activities)	Enjoyment and preference for active PA significantly correlated with athletic competence scores
			“Recreational participation – the types of activities they tend to engage in and to prefer, who they do them with, how much they enjoy their participation and the extent to which their participation takes place at home or is community-based”		Intensity and preference of active PA participation significantly differed as a function of gender. Preferences also significantly differed by age^b^
			“Children’s participation – that is their involvement in life situations such as…”		

Lauruschkus et al. (33)	*Research in Developmental Disabilities/*Sweden	CP (*n* = 364, M = 12.0)	*Participation in PA*: “PA defined as any voluntary bodily movement, produced by skeletal muscles, that requires energy expenditure. Participation in PA (in addition to performance, i.e., what one actually does) was defined as involvement in life situations, including physical, social, and self-engagement in activities”	*Structured Questionnaire* administered by trained professional: participation in PE at school (yes/no) and mean frequency of active participation in physical activities during the preceding year	Majority of participants actively participated in PE (87%), with active participation in PE 1–2 times per week reported by 74% of participants. Frequency of participation was observed to be a factor of age and level of functional impairment emerged as factors of

Lauruschkus et al. (34)	*Disability and Rehabilitation/*Sweden	CP (*n* = 16, M = 9.0)	*Sports participation*: “Participation is defined as involvement in life situations according to the ICF. For children with [CP] the attributes of child, family, environment and physical and social conditions, as well as the degree of self-engagement, are crucial with regard to participation”	*Interviews and Focus Group*: content analysis	Categories/Sub-Categories: (1) Facilitators, “Being physically active because…” (a) enjoying the feeling, (b) being capable, (c) feeling of togetherness, (d) being aware it is good for me, (e) using available opportunities; (2) Barriers, “Being physically active but…” (a) getting tired and experiencing pain, (b) something being wrong with my body, (c) being dependent on others, (d) not being good enough, (e) missing available opportunities

Marquis and Baker (35)	*Research in Developmental Disabilities/*United States	DD and TD (*n* = 63/98, M = 6.0)	*Sports participation; Participation in (physical) activities*: sports broadly defined to include any physical activity reported, from organized team sports to leisurely physical activities Operational definition of ‘sport participation’ *not* provided	*Child Behavior Checklist for ages 6–18 (CBCL)*: number of sports, number of consistent sports and highest relational sport (coded for autonomy/relatedness continuous scale based on Self-Determination framework)	Sports participation was observed to be a factor of (a) child’s delay status and (b) maternal education and hours of work, for all indices of participation. Age was no longer a significant predictor among children older than 8 years

Mâsse et al. (36)	*Research in Developmental Disabilities/*Canada	NDD/D and CMC (*n* = 145/180, M = 9.5)	*Participation in (un)supervised PA at school; Participation profile*: “The ICF defines participation as ‘involvement in life situations’ for children it involves participation in educational, social, recreational and physical activities. Participation in a broad range of activities… is thought to be a key indicator of a child’s health, irrespective of disability”	*Participation and Activity Limitation Survey (PALS Survey)*: attendance (do you take part in?) and type of PA (0 = non-participation and 1 = participation)	NDD/D significantly more likely to participate in (un)supervised activities than CMC. Highest participation in PA at school was among 8–11 years old, compared to 12–14 years old, children with milder disabilities, and among families who did not receive familial assistance

Mitchell et al. (37)	*Developmental Medicine and Child Neurology/*Australia	CP (*n* = 102, M = 11.0)	*Participation in (sports and) PA*: operational definition *not* provided	*GT3X + Tri-Axial Accelerometer*: average activity counts/minute, standardized inactive time and MVPA	Average time spent inactive and in MVPA was a factor of gross motor function level, age, and sex

Moola et al. (38)	*Adapted Physical Activity Quarterly/*Canada	CHD (*n* = 13, M = 14.0)	*PA participation*: operational definition not provided	*Assessment of Life Habits (LIFE-H)* recreation domain: ability to participate in recreational task and level of difficulty or assistance required	Activity counts were shown to have a significant but weak correlation with participation scores (*r* = 0.02, *p* = 0.89)
				*Participation and Environment Measure for Children and Youth (PEM-CY)*: frequency of participation in home, school and community	Activity counts were shown to have a significant but weak correlation with frequency of participation in the home (*r* = 0.31, *p* < 0.001), school (*r* = 0.30, *p* < 0.01) and community (*r* = 0.38, *p* < 0.001)
				*Semi-structured interviews* to examine perceptions of PA and sport participation, self-efficacy and facilitators and barriers to participation: *thematic analysis*	Themes: (1) Sport and PA^c^ not a valued pursuit in relation to other, more important activities that youth engaged in; (2) low self-efficacy toward being physically active; (3) instrumental relationship with sport – participation not only important but instrumental for health benefits; (4) PA participation negotiated within prevailing experience of cardiac disease related to fatigue

Must et al. (39)	*Journal of Physical Activity and Health/*United States	ASD and TD (*n* = 53/58, M = 6.0/6.7)	*Participation in PA*: operational definition not provided	*Parent-Completed Questionnaire* on perceived child/family, social, and community barriers to child’s participation in PA: *total number of barriers in each category and overall*	Number of barriers to PA significantly differed between children with and without ASD. Approximately half of parents identified 6 or more barriers to PA

Shields and Synnot (40)	*BMC Pediatrics/*Australia	CWD Professional stakeholders (*n* = 23/20, M = 13.9)	*Participation in PA*: operational definition *not* provided	*Questionnaire* on participation in (un)organized PA: total number of different activities per year; average number of hours per week spent in PA during the last year	Total number of barriers was inversely correlated with average number of hours in PA (*r* = −0.27, *p* = 0.5) and diversity of PA type per year (*r* = −0.24, *p* = 0.08)

				*Focus Groups*: thematic analysis	Themes: (1) similarities and differences exist between children with and without disabilities; (2) people make a difference; (3) one size does not fit all… it is about choice; (4) communication and connections between stake holders
					[See original manuscript for detailed list of sub-themes categorized by barriers and facilitators]

Sit et al. (41)	*Adapted Physical Activity Quarterly/*China	CWD (*n* =237, M = 13.5)	*Sport participation; Sport participation patterns; Participation in PA*: “who participates in sport and why (i.e. demographics, motives, affordances, barriers, and benefits)”	*Structured interviews with questionnaire*: (1) Membership of sport club/organization, Frequency of attendance in sports or PA participation in past year motives for sport participation, motives for non-participation; (2) Desired and undesired sport and PA participation and type, frequency, and venue of desired sport participation	13% reported membership in sport club or organization^d^
			“…defined sport as physical activity for health, recreation, or competition that is perceived by children as fun, health, and goal oriented”		Frequency of sport participation ranged from 1–2 times per week to 1–2 times per month^d^
					83% participated in at least 1 sport, 66% in at least 2, 46% in at least 3, and 33% in 3+. The majority of participation occurred in public or community venues^d^
					Themes: (1) motives: fun, fitness, achievement, friends, competence, praise, non-conformist, told to (2) non-participation: own thing, other leisure/achievements, lack of skills, watch others, no friends, obligation, let down

*^a^CAPE considered direct measure of participation, documenting what a child does, not the child’s competence in performing an activity or the degree of support the child requires to take part*.

*^b^Results used to support clinical and research utility of CAPE and PAC*.

*^c^Youth did not distinguish between sport and PA*.

*^d^Across indices, sport participation was function of gender and disability type*.

Table 2 summarizes the research approach outlined by the included studies to investigate PA participation. It is organized in accordance with Ross et al.’s (23) guidelines, classifying the approach into (a) the guiding framework, (b) the operational definition of PA participation, and (c) aspects of the measurement tools used to capture this key construct.

**Table 2:** **Number of included articles reporting steps of research approach classified as (1) Theoretical guiding framework, (2) Operational definition of key construct – participation and/or physical activity, and (3) Assessment used to measure physical activity participation along a performance and/or involvement dimension**.

Quality criterion	Number of studies (N = 17 total)
**(1) Theoretical framework**	
ICF or ICF-CY	7 (41%)
PAD	2 (12%)
Other	4 (24%)
None	4 (24%)
**(2) Operational definition of**	
**Participation**	
“(children’s) *participation* is involvement in life situations, such as physical activities”	5 (29%)
“aspects of *sports participation* include frequency [and duration]”	2 (12%)
**Physical activity**	
“*physical activity* is defined as voluntary movement, produced by skeletal muscles, that results in energy expenditure”	2 (12%)
**None**	8 (47%)
**(3) Assessment of physical activity participation**	
**Performance**	
*Intensity* (i.e., level of exertion)	5 (29%)
*Frequency* of attendance	10 (59%)
*Performance* (i.e., physical ability to execute physical activity)	2 (12%)
**Involvement**	
*Attitudes/opinions* about personal physical activity patterns	2 (12%)
*Diversity* of activity type	4 (24%)
*(Lived) experiences* of participation (e.g., where, why, with whom child engaged in physical activity)	5 (29%)
*Perceptions* of the role of physical activity in child’s life	1 (6%)
*Preferences* and *enjoyment* of physical activity involvement	1 (6%)

*Studies may have been counted more than once within each quality criterion if they met more than one sub-category*.

*ICF, International classification of functioning, disability, and health; ICF-CY, International classification of functioning, disability and health: children and youth version; PAD, physical activity for people with a disability mode*.

The WHO-ICF (10), or the corresponding 2007 children’s version [ICF-CY; (42)], was the primary guiding framework used (*n* = 8, 47%) (13, 29, 30, 32–34, 36, 37). The Physical Activity for Persons with Disability (PAD) framework, a PA-specific rendition of the ICF, was utilized in two articles (29, 40). Alternative frameworks included the Social–ecological model (39), Systems Theory (27), Theory of Planned Behavior (32), and Sports Participation Theory (41). An operational definition of PA participation was not explicitly provided within any of the included literature. Instead, when an operational definition was provided, participation and PA were presented as independent constructs. Participation was defined by approximately one-third of the studies (*n* = 5, 29%) as the “*involvement in life situations*, … *such as physical activity*” (13, 33–36) – a direct quote from the WHO-ICF framework [(10), p. 10] – or stated that “*sports [physical activity] participation falls under the broader ICF term ‘participation’*” (32). Two studies noted “*aspects of sports participation include frequency, duration, and social (or subjective) experiences*” (35, 36). PA was operationally defined by only two studies, with both referencing Caspersen’s [(12), p. 126] 1985’s definition: “*voluntary movement produced by skeletal muscles that results in energy expenditure*” (29, 33). Nearly half of the studies did not provide an operational definition of either participation or PA (*n* = 8, 47%).

In accordance with Ross et al.’s (23) taxonomy of PA measurement, 10 studies (59%) used traditional performance-based measures of PA. This included outcomes of percent time in moderate-to-vigorous PA [i.e., *intensity*; (30)], number of PA opportunities attended in the last 6 months or year [i.e., frequency; (26, 33, 35, 36)], or physical ability to execute PA tasks [i.e., motor *performance*; (31)]. Three studies used an accelerometer to capture this data, whereas the remaining eight studies used PA-oriented surveys, daily logs, or interviews.

Physical activity was measured along an alternative involvement dimension of participation within 14 studies (82%). Assessments used included the *Children’s Activity, Participation and Enjoyment* (CAPE) measure (13), the *Child Behavior Checklist* [CBCL; (35)], and the *Participation and Activity Limitation Survey* [PALS; (36)]. Emergent themes from questionnaires and interviews included questions of children’s *experiences* during PA (e.g., where, why, and with whom; 29% of studies), the number of different types of PA opportunities they attended (i.e., diversity; 29% of studies), and their *attitudes or opinions* about personal PA (12% of studies) and their *perceptions* or level of *enjoyment* during PA (12% of studies). Of the 13 studies that used an involvement-oriented measure of PA participation, seven (41%) concurrently assessed PA participation with a performance-oriented measure and either referred to the ICF/ICF-CY or explicitly defined participation as a health construct.

Included studies were primarily descriptive research designs and aimed to (a) describe the perceptions and experiences of PA participation among disabled children and key stakeholders (13, 27–29, 31–34, 38, 41), (b) identify barriers and facilitators to PA participation (28, 29, 31, 34, 35), and/or (c) compare PA participation across groups of disabled children and in relation to non-disabled peers (13, 26, 30, 35, 36, 39). Key outcomes associated with these aims are presented in Table 1.

## Discussion

The primary objective of this mini-review is to critically examine current conceptual and methodological approaches to examining PA participation as an index of health among disabled children. The spike in publications inclusive of this term in 2015 indicates a growing interest in this phenomenon. As anticipated, discussion of PA participation is predominately occurring within fields of psychology and medical rehabilitation research [e.g., Ref. (8, 9, 11)]. The descriptive nature of the included studies, aimed at identifying *what PA participation looks like* and *what it means* to disabled children, indicates our understanding of this construct as an index of health is still in its early stages.

We found two patterns for how researchers approached the conceptualization of PA participation. The first approach framed PA as a context in which participation occurs. Articles using this approach started with the WHO-ICF/ICF-CY definition of participation – “involvement in life situations” [(10), p. 5]. This was followed by the identification of PA as an important context in which children participate. For example:

The [ICF] defines participation as involvement in life situations and for children it involves participation in educational, social, recreational and [PA] [(36), p. 2246]

…children’s participation – that is their involvement in life situations such as personal maintenance, mobility, social relationship, home life and education. Existing measures vary in scope, with some focusing on children’s [PA], others on play, and some including school-based activities [(13), p. 29]

This approach contextualizes health behaviors within specific, child-relevant settings. While it provides a descriptive profile of what, where, with whom, and how often children engage in PA, it does not directly map these behaviors onto health outcomes. For example, Sit et al. (41) concluded that the number of sports disabled children attended was associated with their degree of functional impairment. There are challenges, however, with translating this to a scale of health, because we know little about the children’s physical and psychosocial experiences while engaged in sport. Similarly, *differences in* frequency or intensity between age groups, gender, or disability status (13, 26, 33, 35–37, 41) are difficult to use as a direct *comparison* of health status across groups. For example, children of varying disability status may report low PA intensity due to physical impairments but equitable perceptions of communal involvement (43). However, this approach offers an important first look at the factors and mechanisms associated with PA participation that may be unique to disabled children.

The second approach framed PA as a multi-dimensional construct, with participation included as one of its dimensions. Compared to the first approach, PA served more directly as an index of physical and psychosocial health. It was described in terms of both performance outcomes (frequency and intensity of physical involvement in PA) and participation outcomes (social experiences, perceptions of inclusions or engagement, enjoyment). For example:

For this study, [PA] consists of both [PA] in activities of daily life, such as (hand) biking to school or active play, and participation in (un)organized sports. It is defined as any bodily movement, produced by skeletal muscles, that results in energy expenditure [(29), p. 2]

…empirical attention toward different aspects of sports participation (e.g., frequency and social nature of sport) should be expanded upon for a more comprehensive understanding of sports participation difference [between children with and without disabilities] [(35), p. 46]

This approach provides a foundation for mapping descriptive measures of PA participation to health status for disabled children. The connection between PA participation behaviors and health status is made transparent by the use of qualifiers. For example, Capio et al. (30) measured PA participation *level* (i.e., intensity) as an index of physical function and cardiovascular health. Harvey et al. (31) measured movement skill level as an index of PA participation *competence* or *performance*. King et al. (13) and Mâsse et al. (36) measured PA participation *profiles* to capture more global psychosocial *experiences* of children (enjoyment, perceptions of inclusion, satisfaction). From this framework, subsequent research can begin to translate descriptive PA participation behaviors onto scales of health. This effort would facilitate the identification of “levels” and “experiences” that put children at risk for poor health outcomes throughout life. It would further inform key aspects of PA participation that need to be supported throughout childhood to promote healthy development.

Drawing from the broader discussion of participation in the literature (8–11, 23, 44–48), we offer a working definition of PA participation as it pertains to active, health-associated behaviors:

Physical activity participation describes “experiences in physically demanding movement, sport, game, or recreational play that results in energy expenditure and perceptions of communal involvement.”

It can be qualified by:

(1) Level: *frequency of attendance and intensity of physical exertion* [e.g., Ref. (23, 48)].
(2) Quality of experience: *self-perceived feelings of social inclusion, enjoyment, self-efficacy, and satisfaction* [e.g., Ref. (47)].
(3) Overall profile: *extent to which a child’s level of participation matches their expectation for a quality experience* [e.g., Ref. (11, 43)].

Our emphasis on qualifiers aligns with contemporary works advocating that “*participating in a sport activity for a child with a disability cannot be restricted to health and physical outcomes because participation does not only refer to taking part in an activity, particularly for children with disabilities*” [(20), p. 748]. The predominant use of interviews in the studies reviewed suggests that self-report is the preferred method for capturing PA participation *profiles* of disabled children.

Future efforts are needed to translate our working definition of PA participation into inclusive assessments that map onto indices of health in childhood. Framing PA as a context in which participation occurs was the necessary first step in understanding PA as a dynamic health experience. When framed in this way, we effectively say that PA occurs in life situations (which is self-evident) and is a kind of participation (i.e., we are physically active by participating in PA). The use of participation thereby serves as a filler word and is not, in and of itself, representing a measurable health behavior. We therefore recommend, that moving forward, researchers adopt the term PA *engagement* when referring to PA as a context for participation and measuring physiological behaviors and outcomes (i.e., energy exertion, attendance frequency), synonymous with traditional discussion of PA levels (49, 50). PA *participation* can then serve to represent a broader health experience associated with dynamic child-environment interaction (i.e., *self-perceived feelings of social inclusion, enjoyment, self-efficacy, and satisfaction*). Differentiating PA engagement and PA participation consistently within health-related fields, and approaching PA participation as a measurable construct are further required to support effective assessment of the health status among disabled children.

## Author Contributions

Substantial contributions to the conceptualization and development of this mini-review were made by the primary authors (SR, KB, SL, and LC). HT and JF made substantial contributions to the acquisition and synthesis of data. The manuscript was initially drafted by SR, with all authors contributing to the interpretation of results, development of the written manuscript. The final manuscript was approved by all authors.

## Conflict of Interest Statement

The authors declare that the research was conducted in the absence of any commercial or financial relationships that could be construed as a potential conflict of interest.
