# Supplementary figures and images for: Physical Activity Participation of Disabled Children: A Systematic Review of Conceptual and Methodological Approaches in Health Research
Source: Front Public Health. 2016 Sep 5;4:187. doi: 10.3389/fpubh.2016.00187 (PMC5011128; doi:10.3389/fpubh.2016.00187)

APPENDIX A

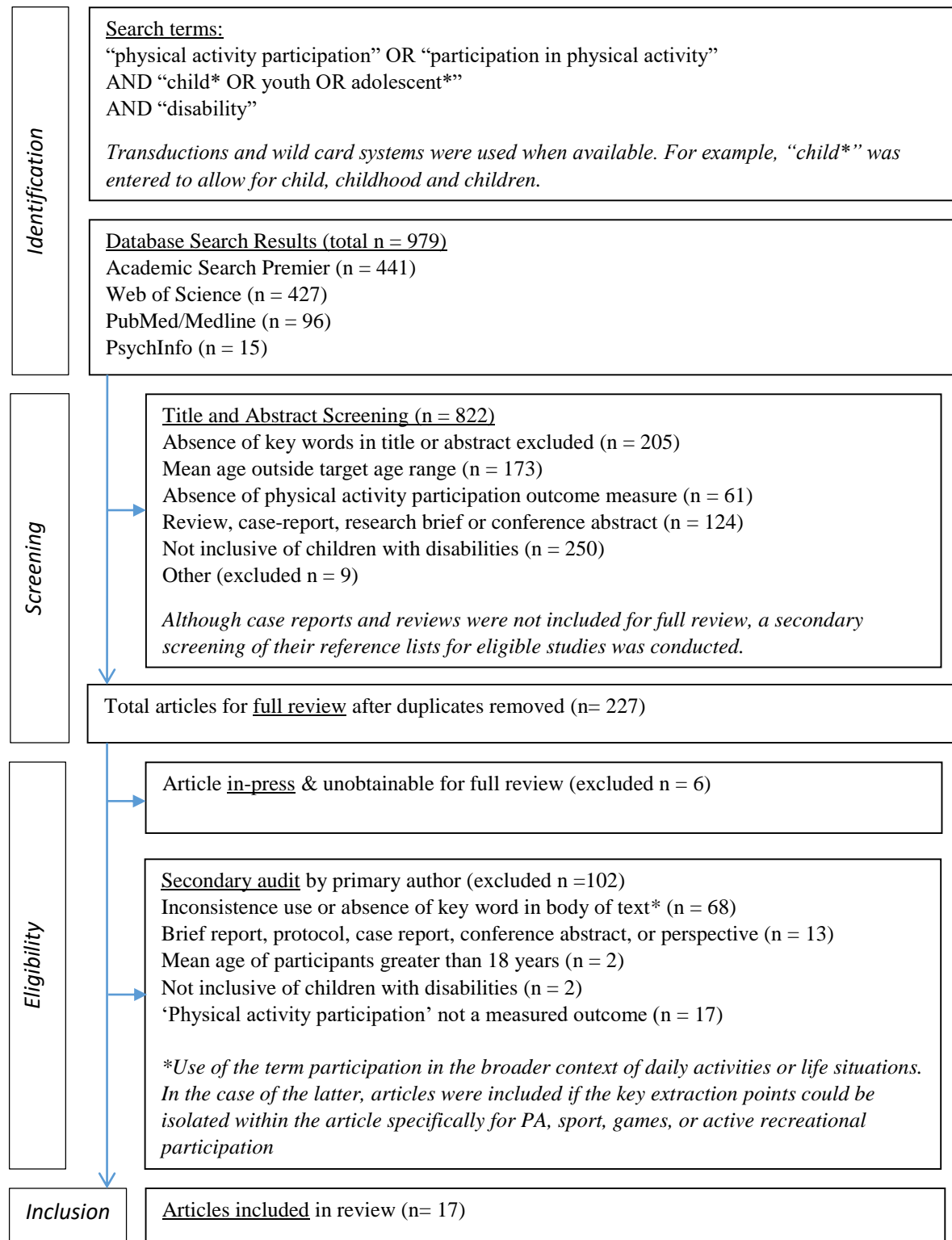

Supplement: Supplementary file 1 [file table_1.pdf]
